# Pharmacokinetics of PEGylated Recombinant Human Erythropoietin in Rats

**DOI:** 10.1155/2014/918686

**Published:** 2014-01-09

**Authors:** Xiaohan Cao, Zhiyong Chen, Zhuoran Yu, Yonghong Ge, Xianyin Zeng

**Affiliations:** ^1^Isotope Research Laboratory, Sichuan Agricultural University, Ya'an 625014, China; ^2^Chengdu Institute of Biological Products, Chengdu 610023, China

## Abstract

rHuEPO plays a central role as chemicals for the treatment of many diseases. Due to its short half-life, the main aim for this pharmacokinetic study is to investigate a newly developed PEG-rHuEPO with large molecular weight in SD rats. After a single intramuscular administration of different doses of 125I-PEG-rHuEPO, pharmacokinetic parameters, tissue distribution, and excretion were analyzed. In *in vivo* half-life time measured after 125I-PEG-rHuEPO administration at the doses of 1, 2, and 3 *μ*g/kg, *t*1/2*α* was 1.90, 1.19, and 2.50 hours, respectively, whereas *t*1/2*β* was 22.37, 26.21, and 20.92 hours, respectively; at 8, 24, and 48 hours after intramuscular administration, PEG-rHuEPO was distributed to all of the examined tissues, however, with high concentrations of radioactivity, only in plasma, blood, muscle at the administration site, and bone marrow. Following a 2 *μ*g/kg single intramuscular administration, approximately 21% of the radiolabeled dose was recovered after almost seven days of study. Urine was the major route of excretion; 20% of the administered dose was recovered in the urine, while excretion in the feces was less than 1.4%. Therefore, this PEG-rHuEPO has potential to be clinically used and could reduce frequency of injection.

## 1. Introduction

Erythropoiesis, the formation of red blood cells from multipotent stem cells in the bone marrow, is an exquisitely regulated process in which the glycoprotein hormone erythropoietin (EPO), which is primarily produced in either adult kidney or fetal liver [1], plays a role [2] as the major humoral regulator of red cell production [3].

This functional pleiotropy of EPO has led to its clinical use for the treatment of several diseases [4–6]. Although obviously time is required to understand some parts of the mechanism, with revelations of novel physiological functions and tissue-specific regulation clearly, there are likely to be more surprises in clinical usage of recombinant human erythropoietin (rHuEPO) along the way [7].

Not surprisingly, rHuEPO is amongst the top selling pharmaceutical products worldwide [7]. The first commercial product epoetin has a plasma half-life of only 6–8 h [7] in humans. The second-generation erythropoietic agent [8] has an approximately 3-fold longer plasma half-life, as long as the third-generation, which has been investigated in phase II [9] clinical trials. Another new erythropoietin derivative with successfully prolonged half-life has been reported recently [10]. However, rHuEPO must be administered relatively frequently, in general 3 times a week either intravenously or subcutaneously due to the short half-life of these agents. Consequently, it is difficult to administer epoetin [11] for patients who need long duration of therapy.

Though applications of new-type HuEPO have increased in recent years, efforts in enhancing the serum half-life of protein medicines need to be improved. So far, several strategies have been developed, including increasing the effective molecular weight of a protein, and therefore reduce renal clearance [12]. The purpose of our experiments was to investigate the pharmacokinetics of PEGvlated rHuEPO (PEG-rHuEPO) with remarkably large molecular weight in rats.

## 2. Materials and Methods

### 2.1. Chemicals and Radiolabeling

Our large-rHuEPO was provided by Chengdu Institute of Biological Products (CDIBP). ^125^I-Sodium iodine, with the radiochemical purity >99.9%, was purchased from NEN Life Science Products, Amersham Inc. Human serum albumin (HSA) was purchased from Chengdu Institute of Biological Products. All other chemicals and reagents were analytical grade and commercially available.

Iodination of PEG-rHuEPO was performed by the two-phase chloramine-T method using ^125^I as described previously [13]. The radiochemical purity and specific radioactivity were over 96% and 28353 dpm/ng, respectively, as determined by trichloroacetic acid (TCA) precipitation. There was no loss of biological activity after labeling demonstrated by reticulocyte counting method. Injection buffer was composed of 100 mM NaCl, 20 mM citrate buffer (pH7.0), and 2.5 mg/mL HSA.

### 2.2. Animals

35 Sprague-Dawley (SD) rats, weighing from 180 to 220 g, were purchased from Sichuan University Animal Experiment Centre. The animals were weighed and randomly divided into three groups: a single intravenous administration of ^125^I-PEG-rHuEPO at 1, 2, and 3 *μ*g/kg bodyweight (delivered as a solution in 0.4 mL injection buffer) as group I (5 rats per dose); a single intramuscular administration of 2 *μ*g/kg dose of ^125^I-PEG-rHuEPO (diluted to 0.4 mL with injection buffer) as group II; 5 rats were sacrificed by decapitation at 8, 24, and 48 h after dose administration, respectively; 5 rats equipped with jugular vein catheters received a 0.4 mL intravenous bolus of ^125^I-PEG-rHuEPO at the dose of 2 *μ*g/kg as group III.

### 2.3. Plasma Pharmacokinetics Studies

Two heparinized polyethylene cannulas were placed in both femoral artery and vein of rats for intravenous administration and blood collection. Blood (100 *μ*L) was withdrawn from rats (*n* = 5 per concentration of administration) through an arterial cannula into a heparinized Eppendorf tube at 2, 5, 10, and 40 min and 1, 2, 4, 6, 8, 24, 48, and 72 h p.i. (after inoculation) and stored at 4°C for further analysis. At the time of analysis, plasma was prepared by centrifugation of blood at 3000 rpm for 10 min. 185 *μ*L water and 15 *μ*L plasma were added to another centrifuge tube and incubated for 10 min after stirring, followed by adding 200 *μ*L 20% trichloroacetic acid (TCA). The reactive mixture was allowed to stand for at room temperature and centrifuged at 3000 rpm for 30 min. The radioactivity associated with the insoluble pellet was determined in duplicates. Plasma concentration was calculated accordingly and fitted to the tow-compartment model. Pharmacokinetic parameters were estimated from the plasma concentration data using 3P97 program developed by Chinese Pharmacological Society (CNPHARS).

### 2.4. Tissue Distribution Studies

After the rats were sacrificed (*n* = 5 at each time points, resp.), the following tissues and organs were immediately collected to determine radioactivity: plasma, blood, red blood cells, brain, thyroid, heart, liver, pancreas, spleen, lung, lymph nodes, kidneys, adrenal gland, dorsal muscle, muscle at the injection site, urinary bladder, stomach, small intestine, large intestine, and bone marrow. Whole tissues were weighed and subsequently measured directly by gamma counter. Large tissues were grinded and partly analyzed following the measuring of total weight. Trunk blood was collected in a heparinized tube, 100 *μ*L of which was analyzed for gamma emission. The rest was processed for plasma by centrifugation; 100 *μ*L plasma was collected for gamma counting. Red blood cells were extracted from another 100 *μ*L plasma and washed by water for gamma counting.

### 2.5. Excretion Studies

A small amount of physiological saline was injected intravenously immediately after administration. The rats (*n* = 5) were housed individually in silicone-treated metabolic cages. Urine and feces were collected at 24 h intervals for eleven consecutive days after dose administration. For solid and liquid samples, an aliquot (7 g) of homogenized feces and an aliquot (1 mL) of urine were used for measuring radioactivity.

### 2.6. Statistical Analysis

The comparison of pharmacokinetic parameters was performed using *Q* test (Newman-Keuls method). Statistical significance was taken as *P* < 0.05.

## 3. Results and Discussion

### 3.1. Plasma Pharmacokinetics

**Figure 1:**
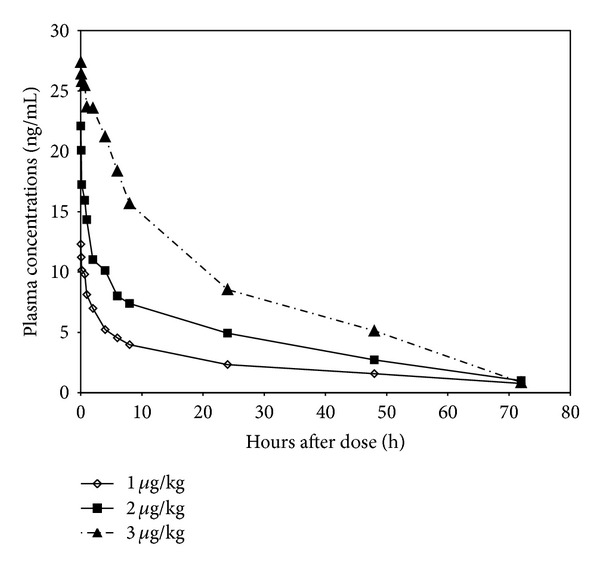
Plasma concentration-time profiles for PEG-rHuEPO after intravenous administration to rats at doses of 1, 2, and 3 *μ*g/kg.

Figure 1 shows the plasma concentration-time profiles of PEG-rHuEPO after intravenous route of administration at doses of 1, 2, and 3 *μ*g/kg. The pharmacokinetic parameters calculated from the data are summarized in Table 1.

Hybrid coefficient B, initial phase half-life (*t*1/2*α*), transport rate constant from circumjacent compartment to central compartment (K12), and area under plasma concentration time curve (AUC) were prolonged with increasing dose. Solely circumjacent compartment to central compartment (K10) fell with increasing dose and at high doses. For hybrid coefficient A, hybrid first-order rate constant *α*, volume of the central compartment (*V*_*c*_), terminal phase half-life (*t*1/2*β*), elimination rate constant from the central compartment (K21), and systemic clearance (CL_s_), peak value appeared following the administration of 2 *μ*g/kg dose. In contrast, hybrid first-order rate constant *β* was the lowest in 2 *μ*g/kg dose group.

Statistical comparisons of individual pharmacokinetic parameters such as *α*, *β*, *V*_*c*_, *t*1/2*α*, *t*1/2*β*, and K21 demonstrated no significant (*P* < 0.05) differences when each parameter was compared for each two of the three test doses. However, significant differences among each two of the three dose groups were seen in two parameters, AUC and B. A significant difference was detected only between the calculated after one dose of 1 *μ*g/kg and the corresponding value resulting from the administration of 2 *μ*g/kg dose. Only between doses of 2 *μ*g/kg and 3 *μ*g/kg, significant differences existed in K12 and CL_s_. 1 *μ*g/kg and 3 *μ*g/kg doses shared the significant differences in transport constant from K10.

*Half-Life of PEG-rHuEPO.* Since serum half-life is identified as a major controlling factor of *in vivo* biological activity of EPO [10], the redesign of rHuEPO to promote longer serum half-life is an important medical goal, leading to reduce the frequency of administration by injection [12]. To enhance the serum half-life, several strategies have been developed. In hyperglycosylated darbepoetin, also known as novel erythropoiesis stimulating protein (NESP), two extra N-linked sialic acid-containing oligosaccharides were incorporated to confer greater metabolic stability *in vivo* [11]. Also, continuous erythropoietin receptor activator (CERA), a large polymer chain, was introduced [8]. Another enhancement for the biological activity of rHuEPO is to provide a “protective vehicle” so as to decrease the rate of elimination. Methods such as microencapsulation and pegylation to rHuEPO have been reported [14]. Lee et al. [10] recently constructed novel EPO derivatives with prolonged half-live times by adding peptides to the carboxyl terminus of EPO without using linkers. Lee also demonstrated that addition of these peptides did not interfere with secretion or receptor binding and significantly increased the *in vivo* half-life of human EPO. Tillmann et al. [15] investigated the efficacy of new erythropoietic agents, with polyethylene glycol (PEG) conjugated proteins in rats with chronic renal insufficiency, which can prolong elimination half-life time and diminish protein immunogenicity. Additionally, the cost and inconvenience associated with chronic frequent parenteral administration of EPO have led a number of investigators to seek ways to deliver proteins orally. A desirable improvement sought for EPO therapy is in the method of drug delivery. A second strategy to obtain orally administered agents is to discover small molecule drugs that retain the full agonist activity of the large protein molecules. However, these research strategies are difficult and highly risky [16].

In this study, we investigated pharmacokinetics of a newly developed long-acting recombinant human erythropoietin (PEG-rHuEPO), concentrating on its characteristic of *in vivo* half-life time. The strategy of increasing molecular weight was employed. Pharmacokinetic data indicated good linear relationship between three dose levels and AUC, with R2 0.9685. More encouragingly, data from intravenous study is sufficient to prove that plasma half-life (*t*1/2*α*, 1.2 to 2.5 h; *t*1/2*β*, 21 to 26 h) of PEG-rHuEPO is significantly prolonged compared with results from previous reports of native and recombinant human EPO in rats, with *t*1/2*α* ranging from 2.0 min to 53 min and *t*1/2*β* from 86 min to 2.7 h [17–19], consistent with the latest publications [20–22]. Hypothetically, it could be expected that our large-rHuEPO under current investigation will give a similar successful performance in the aspect of half-life time.

*Administration Routes. *rHuEPO can be administered either subcutaneously, intravenously, or intraperitoneally. Intraperitoneally (i.p.) administered rEpo produced higher plasma concentrations (1AUC and 1*C*_max_) than did s.c. administration. This is partially due to more rapid absorption after i.p. administration. Correspondingly, the longer absorption time for s.c. (1*T*_max_ and 2*C*_max_) may have contributed to the longer *t*1/2 for subcutaneous injection [23]. Compared with the intravenous route, subcutaneous rHuEPO administration significantly prolongs the increase of serum erythropoietin, thus sustaining the stimulation of erythropoiesis. Furthermore, up to 30% reduction in total weekly rHuEPO administrations on haemodialysis patients could be achieved to maintain the same haemoglobin level when switching intravenous to subcutaneous administration. However, as there is an increased concern of antibody-mediated pure red cell aplasia associated (PRCA) with subcutaneous EPO administration to renal patients, a change in the route of EPO-alpha administration from subcutaneous to intravenous has been recommended [14, 24]. Moreover, it has been reported that subcutaneous rHuEPO can cause pain and local irritation at the injection site. Fewer side effects being reported after intravenous administration and the long-acting character of this large-rHuEPO could to some extent compensate the shorter half-life time of EPO in intravenous treatment; intravenous administration route which was recommended.

### 3.2. Tissue Distribution

**Figure 2:**
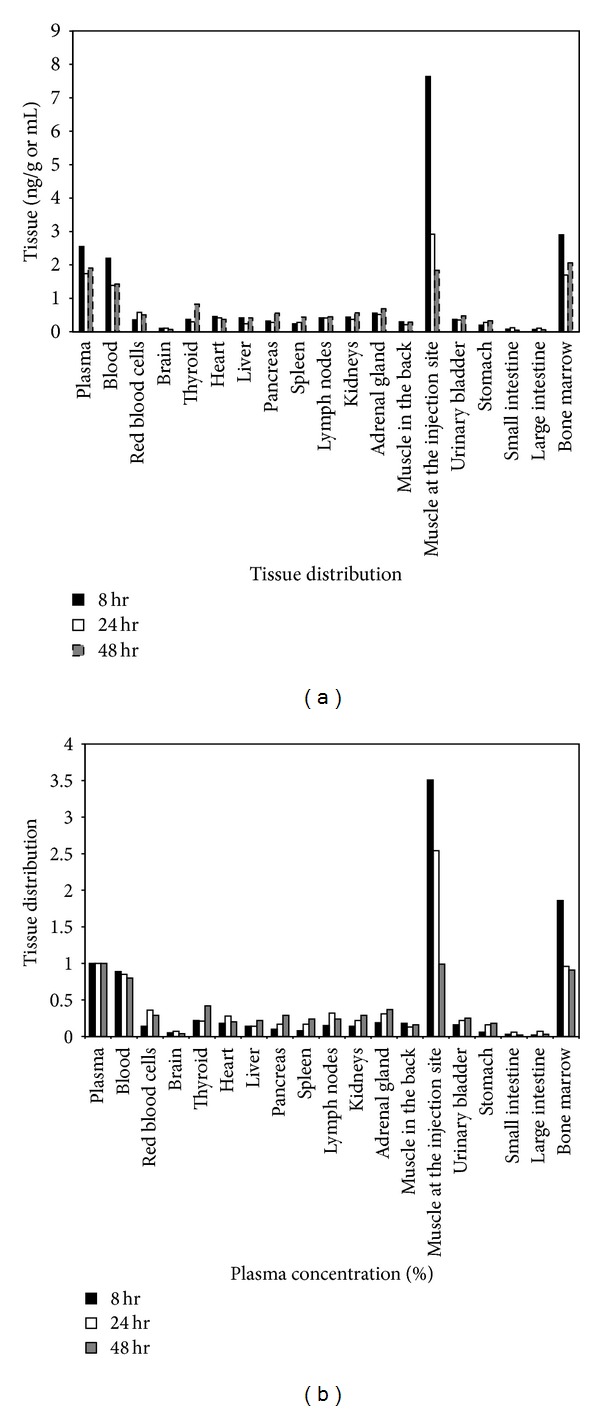
(a) Tissue distribution (ng/g or mL tissue) of PEG-rHuEPO at 8, 24, and 48 hrs after a single intramuscular injection of ^125^I-large-rHuEPO in rats at the dose of 2 *μ*g/kg. (b) Tissue distribution as percentage of plasma concentration at 8, 24, and 48 hrs after a single intramuscular injection of ^125^I-PEG-rHuEPO in rats at the dose of 2 *μ*g/kg.

Figure 2(a) shows the distribution of PEG-rHuEPO in different tissues at 8, 24, and 48 h after administration.  Figure 2(b) shows tissue distribution as percentage of plasma concentration. PEG-rHuEPO was detectable in all tissues analyzed at all-time points. However, except in plasma (2.55 ± 1.08, 1.74 ± 0.70, and 1.91 ± 0.55 ng/mL), blood (2.20 ± 0.91, 1.39 ± 0.36, and 1.43 ± 0.15 ng/mL), muscle at the injection site (7.64 ± 4.49, 2.92 ± 1.45, and 1.84 ± 1.67 ng/g), and bone marrow (2.90 ± 3.20, 1.70 ± 1.55, and 2.06 ± 1.53 ng/g), the tissue/blood ratios of PEG-rHuEPO concentration are remarkably low. The tissues with the lowest exposure were brain (0.10 ± 0.08, 0.11 ± 0.03, and 0.07 ± 0.02 ng/g), small intestine (0.05 ± 0.03, 0.12 ± 0.07, and 0.05 ± 0.03 ng/g), and large intestine (0.07 ± 0.04, 0.11 ± 0.07, and 0.06 ± 0.02 ng/g). Blood to plasma ratio was approximately 0.85 and remained relatively constant over time.

The low distribution of PEG-rHuEPO may be due to its large molecular weight. Favorably, except blood, plasma, and muscle at the injection site, the high concentration of PEG-rHuEPO in bone marrow, which is the main site where EPO carries out its function, indicates its similar *in vivo* activity compared with endogenous EPO. The extremely low concentration of PEG-rHuEPO in the brain can be ascribed to the blood-brain barrier (BBB), for systemic rEpo which crossed the BBB in a dose-dependent manner [23]. Another reason for the low concentration of PEG-rHuEPO in brain is speculated by the normal oxygen environment [23]. In addition, the distribution of PEG-rHuEPO in the bladder is higher than that in the small or large intestine, which confirms, to the results of excretion test, that PEG-rHuEPO is mainly excreted through urine [25].

### 3.3. Excretion

**Figure 3:**
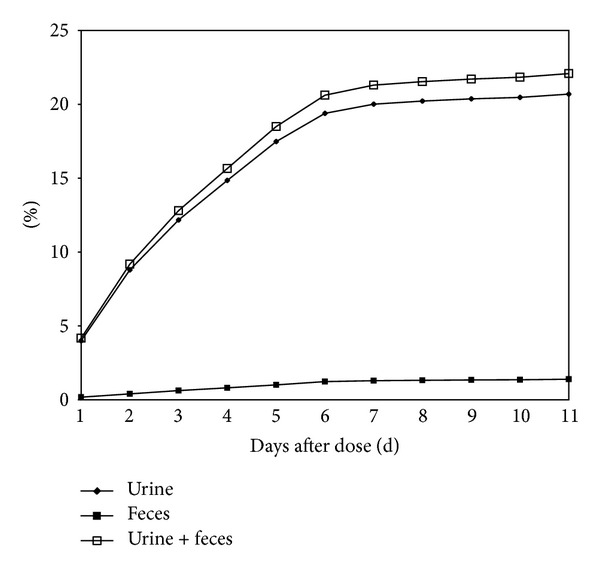
Accumulated percentage of dose excreted in the urine and feces of rats following a single intravenous administration of ^125^I-PEG-rHuEPO at the dose of 2 *μ*g/kg.

Following an intramuscular administration of ^125^I-PEG-rHuEPO, a mean of 21% of the radioactive dose was recovered (Figure 3). Most of the radioactivity was excreted with the urine, the major route of elimination. However, in feces, a mean of less than 1.4% was recovered. Mass balance was generally achieved within the first 48 h of the study.

Only 21% this novel PEG-rHuEPO was excreted mainly thought urine, indicating its high bioavailability and long biological half-life time, which are clinical favorable, and that urine was the major route of excretion. As Jelkmann has reviewed [7], there remains lack of complete knowledge of the main site and mechanism of removal of EPO from the circulation. The kidney may not be a primary site of removal but may contribute to it although several studies investigating EPO pharmacokinetics in normal and in patients with chronic renal failure have not shown differences between the two groups. To a minor degree, EPO may be cleared by the kidneys since hepatocytes can take glycoproteins via the galactose receptor. However, there is evidence to assume that EPO is mainly removed from circulation by uptake into erythrocytes and other cells possessing the EPO receptor. This newly developed PEG-rHuEPO seems undergone the similar degradation fate as described. Accordingly, current new rHuEPO formulations, which contain methoxy-polyethylene glycol to prevent internalization of the drug, thus resulting in prolonged biological half-life time, are tested. This investigated large-rHuEPO may also have employed this strategy.

## 4. Conclusion

Our experiment indicated that the elimination half-life time of this newly developed PEG-rHuEPO is obviously longer than that of the ordinary rHuEPO. In conclusion, this newly developed PEG-rHuEPO has potential to be clinically used and reduce administration frequency. Despite that many attempts have been done, more investigations on *in vivo* potency of EPO remains should be done.

## Figures and Tables

**Table 1:** Pharmacokinetic (PK) parameters calculated from plasma concentrations of TCA-precipitable radioactivity in rats after intravenous administration of ^125^I-PEG-rHuEPO at the dose, of 1, 2, and 3 *μ*g/kg^a^.

PK parameters	Mean ± SD (*n* = 5)		
	1 *μ*g/kg	2 *μ*g/kg	3 *μ*g/kg
*A* (ng/mL)	5.3159 ± 0.3669^b^	8.1045 ± 2.0216^c^	6.8179 ± 0.8774^bc^
*α* (1/h)	0.3940 ± 0.1234	0.6870 ± 0.3366	0.3957 ± 0.2823
*B* (ng/mL)	6.0875 ± 1.0421^b^	9.9443 ± 3.0194^c^	21.6185 ± 1.1005^d^
*β* (1/h)	0.0313 ± 0.0034	0.0273 ± 0.0054	0.0334 ± 0.0033
*V*_*c*_ (mL/kg)	17.7407 ± 2.1606	23.3783 ± 5.5114	21.1202 ± 0.7322
*t*1/2*α* (h)	1.9009 ± 0.5890	1.1983 ± 0.4871	2.5003 ± 1.4982
*t*1/2*β* (h)	22.3658 ± 2.5324	26.2094 ± 4.8848	20.9201 ± 1.9529
K21 (1/h)	0.2269 ± 0.0783	0.3914 ± 0.1934	0.3137 ± 0.2241
K10 (1/h)	0.0547 ± 0.0040^b^	0.0479 ± 0.0092^bc^	0.0421 ± 0.0040^c^
K12 (1/h)	0.1437 ± 0.0447^bc^	0.2749 ± 0.1398^b^	0.0733 ± 0.0589^c^
AUC ((ng/mL)·h)	207.8230 ± 9.5021^b^	373.6992 ± 47.8833^c^	679.9779 ± 70.8449^d^
CL_s_ (mL/kg/h)	0.9640 ± 0.0450^bc^	1.0826 ± 0.1194^b^	0.8904 ± 0.0963^c^

^a^All values are reported as mean ± SD (*n* = 5).

^b, c, d^Means in the same line with different letters of superscript are significantly different (*P* < 0.05).

Abbreviations: *A* and *B*, hybrid coefficients; *α* and *β*, hybrid first-order rate constants; *t*1/2*α* and *t*1/2*β*, initial and terminal phase half-lives; *V*_*c*_, volume of the central compartment; K21, elimination rate constant from the central compartment; K10, transport rate constant from circumjacent compartment to central compartment; K12, transport rate constant from circumjacent compartment to central compartment; AUC, area under plasma concentration time curve; CL_s_, systemic clearance.
